# Musculoskeletal Injuries in Ultra-Endurance Running: A Scoping Review

**DOI:** 10.3389/fphys.2021.664071

**Published:** 2021-03-31

**Authors:** Volker Scheer, Brian J. Krabak

**Affiliations:** ^1^Ultra Sports Science Foundation, Pierre-Bénite, France; ^2^University of Washington and Seattle Children’s Sports Medicine, Seattle, WA, United States

**Keywords:** ultra running, ultramarathon, muscle injury, injury, trail running

## Abstract

Ultra-endurance running (UER) has seen an important increase in participation over the last few decades. Long hours of UER can lead to excessive stress on the body, resulting in musculoskeletal injuries (MSKI). UER is not a uniform sport and events can differ considerably in distance (over 42.195 km), time (e.g., events over 6 h) and multi-day or multi-stage events on various surfaces (e.g., track, on-road, off-road). The aims of this scoping review were therefore: (1) to examine the current evidence of MSKI, providing a synthesis of the most common MSKI by anatomical region and specific diagnosis; (2) categorize MSKI by type of UER activity (competition: time-limited; multi-stage; continuous UER events and training); (3) describe knowledge gaps in the literature and provide advice on potential further research. Our electronic literature search (PubMed, SPORTDiscus, Web of Science) identified a total of 13 studies (9 in competition, and 4 in training). Anatomical site, diagnosis and rate of injuries differ between competition and training as well as between different UER types. MSKI are observed in 18% of multi-stage events (0.7–1.8 injuries/runner and 7.2 injuries/1000 h). Most MSKI involve the lower leg (35.0%), ankle (16.8%), knee (13.1%) and foot (12.6%), with main diagnosis of medial tibial stress syndrome (30.1%) and patella femoral pain syndrome (PFPS; 7.2%). Single, continuous UER events differ between a 1005 km road race with almost all of the injuries due to overuse, with the main anatomical site of the knee (31%), ankle (28%) and lower leg (14%) and main diagnosis of PFPS (15.6%), compared to a 65 km trail race, with 32.8% of MSKI, mainly on the foot [plantar fasciitis (28.6%)], ankle [sprain (28.6%)] and knee. Timed-UER events (injury rate of 2.1 injuries/athlete) observed most injuries on the ankle (36%) and knee (19%), with the main diagnosis of tendinitis of the foot dorsiflexors (30%). Injuries during training most commonly affect, the back (42%), and knee (40%) and bone stress injuries (22%). Main diagnoses include ankle sprain (18%), iliotibial band injury (16%) and Achilles tendinopathy (11%). Future considerations include examining MSKI in different UER events, environments and surfaces, and on larger study populations. Establishing risk factors, examining sex differences and using a standard reporting system of MSKI in UER are also important.

## Introduction

Humans are well suited to running long distances, having evolved as persistence hunters, capable of covering great distances in pursuit of prey (Scheer, 2019). Ultra-endurance running (UER) tests the limits of the human body and has become increasingly popular over the last few decades, with an exponential increase that has slowed slightly since ∼2016 (Scheer, 2019). This increase is in large part due to an increase in female and master athlete (athletes ages > 35 years) participation (Eichenberger et al., 2012; Knechtle et al., 2012; Zingg et al., 2013; Scheer, 2019). In addition, there has been a similar increase in participation of youth athletes (<19 years of age), although numbers are much smaller compared to adults (Scheer and Hoffman, 2019; Scheer et al., 2020b, c). In 2019 alone, over 669,000 runners finished an UER event and there were over 7000 UER events hosted around the world (DUV, 2019). The exception of course, was 2020, with a significant reduction in UER participation and events, due to the global COVID-19 pandemic (Scheer et al., 2021).

Ultra-endurance running is not a uniform sport but can be defined as a broad category with different types of running activities, such as running events by distance (e.g., any distance in excess of the standard marathon distance of 42.195 km), time (timed- UER, e.g., any events over 6 h) and multi day/multi-stage events (distance or timed events over several days or stages) (Scheer et al., 2020a). UER events can be held on various surfaces (e.g., track, on-road, off-road) (Scheer et al., 2020a) and in extreme challenging environments, putting additional strain on the human body (e.g., extreme cold, altitude, mountain, desert, heat and jungle) (Knoth et al., 2012; da Fonseca-Engelhardt et al., 2013; Gill et al., 2015; Scheer and Murray, 2015; Dawadi et al., 2020; Suter et al., 2020). The most popular race distances are those of 50 km, 100 km, and 100 miles (Cejka et al., 2014; Scheer, 2019; Knechtle et al., 2020), but can also include distances in excess of 1000 km (Fallon, 1996; Schütz et al., 2012; Scheer et al., 2020a), whereas time-limited events often include 6, 12, or 24 h events, with some lasting several days (Hutson, 1984; Bishop and Fallon, 1999; Scheer et al., 2020a). Multi day/multi-stage events are often held in extreme environments, and athletes often need to carry their provisions, resulting in additional weight while running as a further challenge (Krabak et al., 2011; Knoth et al., 2012; Dawadi et al., 2020; Scheer et al., 2020a).

Long hours of UER can lead to excessive stress on the musculoskeletal system, and potentially musculoskeletal injuries (MSKI) (Scheer and Murray, 2015). Average training loads in UER are between 66–83 km/week in adults and around 57 km/week in youth athletes (Kłapciñska et al., 2013; Scheer et al., 2018, 2020d; O’Loughlin et al., 2019). These training demands can lead to overuse injuries, especially when the load exceeds the adaptive mechanisms, affecting predominantly the lower limbs (e.g., patellofemoral pain syndrome, medial tibial stress syndrome, Achilles tendinopathy) (Khodaee and Ansari, 2012; Krabak et al., 2013, 2014; Scheer and Murray, 2015). Acute injuries are less common but may impact race performance (Khodaee and Ansari, 2012; Krabak et al., 2014; Scheer and Murray, 2015).

Musculoskeletal injuries in running have been defined as “running-related (training or competition) musculoskeletal pain in the lower limbs that causes a restriction on or stoppage of running (distance, speed, duration, or training) for at least 7 days or 3 consecutive scheduled training sessions, or that requires the runner to consult a physician or other health professional” (Yamato et al., 2015b), however, definitions of MSKI in UER vary across studies, which make comparisons difficult. Some defined MSKI as a disability resulting in a medical encounter (Krabak et al., 2011; Vernillo et al., 2016), or affecting performance (Bishop and Fallon, 1999). The severity of an injury had been described as severe, if it did not improve with rest (Hutson, 1984), major, resulting in race withdrawal, or minor, when the runner was able to continue the race (Krabak et al., 2011; Vernillo et al., 2016).

It is well established that physiological demands within this broader spectrum of UER may vary, depending on event type (e.g., distance, surface, and elevation changes) (Davies and Thompson, 1986; Bassett and Howley, 2000; Millet and Millet, 2012; Scheer et al., 2020a). For example, the fractional utilization of VO_2_max in a 24 h race is between ∼40–50% (Millet et al., 2011), whereas shorter races of 6 h duration can be run at ∼70% of VO_2_max (Giovanelli et al., 2017). If these higher running intensities in UER increase the risk of MSKI is currently unknown. Similarly, UER with large elevation changes, especially prolonged downhill running sections, place particular demand on the musculature with prolonged eccentric muscle action, leading to increased release of muscle enzymes (creatine kinase) and muscle damage (Dewolf et al., 2016; Giandolini et al., 2017; Vernillo et al., 2017). Such variability makes it likely that MSKI and injury rates will also be affected by the different types of UER events, like timed-events, multi-day events and continuous UER events. Similarly, injury rates and diagnosis of MSKI during competition and training may also vary, as during competitions athletes typically push themselves to the limit. Several review articles of illness and injuries exist about ultramarathon running, however, they have not specifically reviewed MSKI in different types of UER events and analyzed MSKI in competition and training (Khodaee and Ansari, 2012; Lopes et al., 2012; Krabak et al., 2013, 2014; Hoffman et al., 2014; Knechtle and Nikolaidis, 2018a, b). Proper treatment of injuries and illnesses in UER is important for avoiding long-term issues (Krabak et al., 2014) and therefore it is important to examine and summarize MSKI within these broad categories of UER during competition and training.

The aims of this scoping review were therefore: (1) to examine the current evidence of MSKI, providing a synthesis of the most common MSKI by anatomical region and specific diagnosis; (2) categorize MSKI by type of UER activity (competition: time-limited; multi-stage; continuous UER events) and training); (3) describe knowledge gaps in the literature and provide advice on potential further research.

## Materials and Methods

This review is based on the recommendations for scoping reviews, with the purpose of identifying and mapping the available evidence and identifying knowledge gaps (Armstrong et al., 2011). As such we used a broad research question, for example: what are the musculoskeletal injuries in UER? What are the different anatomical distributions and incidence/prevalence of MSKI in UER competition and training? Our aim was to review the existing literature, summarize those findings, identify knowledge gaps, as done in previous scoping reviews (Armstrong et al., 2011; Granacher et al., 2016). The review considered scientific papers that investigated MSKI in UER. UER is defined as a broad category with different types of running activities, such as running events by distance in excess of 42.195 km (standard marathon distance), timed-events over 6 h durations, and multi-day or multi stage running events, on all surfaces (road, off-road, track) and terrains (Scheer et al., 2020a). All studies that examined MSKI in UER were included irrespective of participants age and/or sex, however, detailed information of MSKI according to sex or age was not available in the majority of studies, therefore a comprehensive breakdown of MSKI according to these parameters was not possible. However, those studies that did provide specific results on age and/or sex aspects were highlighted in the respective sections in the discussion (Micklesfield et al., 2007; Scheer et al., 2020d).

**FIGURE 1:**
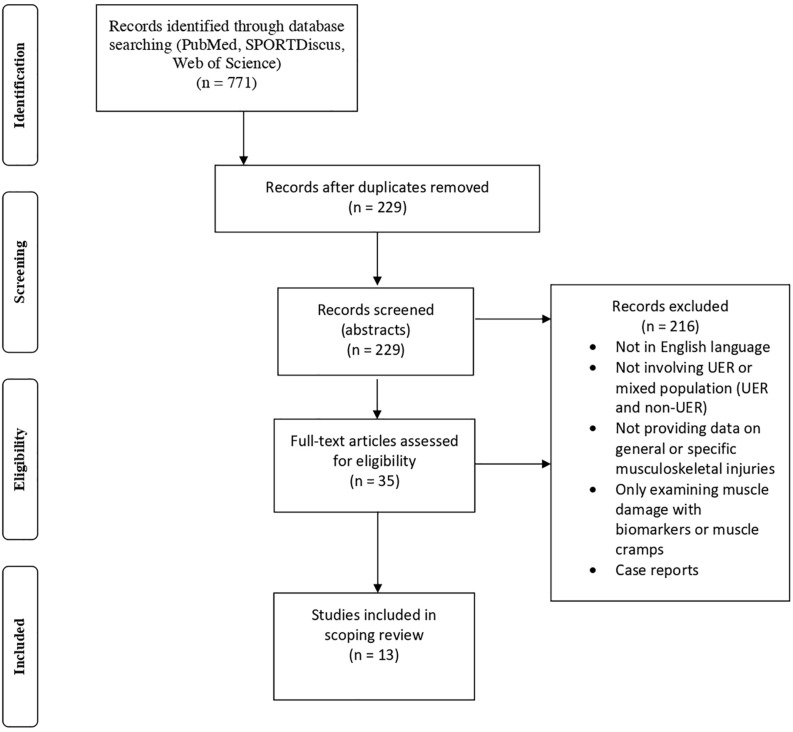
Flow chart of article selection process [adapted from Moher et al. (2009)].

An electronic literature search was performed using different databases (PubMed, SPORTDiscus, Web of Science) from January 1st, 1984- September 30th, 2020. The following search terms were used: ‘ultra endurance running’ or ‘ultra running’ or ‘ultramarathon’ or ‘trail running’ or ‘ultra trail running’ and ‘injury’ or ‘musculoskeletal injury’ or ‘muscle injury.’ We identified a total of 771 studies meeting our initial search criteria. Figure 1 gives an overview of article selection process in accordance with PRISMA (Moher et al., 2009). After removal of duplicates, the abstracts of the remaining 229 studies were reviewed. Only studies written in the English language were considered. Studies that did not examine MSKI in UER, did not provide any data on general or specific MSKI, provided data on mixed population (e.g., different running populations including UER and non-UER) were excluded. Similarly, studies that only examined muscle damage from biochemical markers or muscle cramps, as well as case reports were not included in this review. Studies on blisters, dermatological or other illnesses and injuries were excluded. A total of 35 full texts were assessed for eligibility and after review, a total of 13 studies met our inclusion criteria and were therefore included in the review. All abstracts and manuscript were reviewed by the two authors.

The definitions of MSKI varied across the different studies, with some providing no specific definitions (Scheer and Murray, 2011; Graham et al., 2012; McGowan and Hoffman, 2015; Dawadi et al., 2020), whereas other defined MSKI as a disability resulting in a medical encounter (Krabak et al., 2011; Vernillo et al., 2016), or affecting performance (Bishop and Fallon, 1999). All studies reporting MSKI during a race were either documented by the attending medical team (through self-referral/self- reporting system via medical encounters) or by a routine daily medical assessment questionnaire (Hutson, 1984; Fallon, 1996; Bishop and Fallon, 1999; Krabak et al., 2011; Scheer and Murray, 2011; Graham et al., 2012; McGowan and Hoffman, 2015; Vernillo et al., 2016; Dawadi et al., 2020). Studies examining MSKI in training studies defined injuries as either an athlete reporting the diagnosis based on a health care provider encounter or through athlete self-assessment (Micklesfield et al., 2007; Hoffman and Krishnan, 2014; Malliaropoulos et al., 2015; Scheer et al., 2020d).

## Results

The main findings of our review were: (i) MSKI in UER are common, mostly affecting the lower limbs and are of overuse in nature; (ii) MSKI differ between competition and training, with multistage events, predominantly affecting the lower leg, foot, and knee, while timed events mainly affect the ankle, Achilles tendon, and knee; (iii) Short continuous UER events off-road have the highest incidence of MSKI, mainly affecting the foot and ankle, while long continuous UER affect the knee and ankle; (iv) During training back, knee and bone stress injuries are common. Detailed description of MSKI incidence (rate of occurrences of new cases) and/or prevalence (number of MSKI at a particular time) are provided. The manuscript is organized accordingly describing the different pertinent research sections.

Thirteen studies examined MSKI in UER and are included in this review. Table 1 and Table 2 provide detailed information about the study, participants (age and sex), and MSKI. Nine studies (Table 1) provide information from UER races/competitions (total *n* = 723 participants), while four studies (Table 2) provide information from MSKI mostly during training of UER athletes (total *n* = 1606 participants). Detailed information of MSKI according to sex or age was not available in the majority of studies, therefore a comprehensive breakdown of MSKI according to sex or age was not possible. One study (Micklesfield et al., 2007) specifically examined bone stress injuries in female UER, and another MSKI in youth athletes (Scheer et al., 2020d) and for those studies results by sex and age were discussed in more detail in the appropriate sections. The vast majority of MSKI involve the lower limb (Fallon, 1996; Krabak et al., 2011), and are generally minor across all types of UER (Fallon, 1996; Bishop and Fallon, 1999; Krabak et al., 2014). MSKI are predominantly overuse in nature (e.g., tendinopathies), even during competitions, with incidences in the region of between 98–100% (Fallon, 1996; Bishop and Fallon, 1999), demonstrating the exceptional demand on the musculoskeletal system during prolonged running, in contrast to ‘true’ acute injuries (e.g., muscle strains) that may be expected during competition in other, more explosive sports (Huxley et al., 2014; Krabak et al., 2014; Scheer and Murray, 2015).

**TABLE 1:** Studies investigating musculoskeletal injuries (MSKI) during competition.

Study	Competition/Race	Study design	Participants	General observations	Specific injuries	Non-finisher (MSK injury)	Definition	Additional observation
Hutson, 1984	Track; 6-day race (time-limited) (Charles Rowell Six Day Race, Nottingham, United Kingdom)	observational, prospective	21 (19 men, 2 women; average age 41)	27 MSKI, all overuse	Tendinitis of the foot dorsiflexors 29.6% (severe 18.5%), patellar tendinitis 18.5% (severe, 3.7%), Achilles tendinitis 18.5%, psoas bursitis 11.1% (severe, 3.7%), PFPS 7.4% (severe 3.7%) MTSS 10.1%, gastrocnemius strain severe 3.7%	1 (4.8%) Achilles tendinitis	Self-reported medical encounters to medical team; classification: severe, did not improve with rest	Winner covered 576 miles, 13 athletes passed 400 miles, direction of track was changed every 12 h
Fallon, 1996	Road; 1005 km (Westfield Sydney to Melbourne, Australia)	observational, prospective	32 (average age men 38, women 43; number of male/females not given)	64 MSKI (63 overuse injuries); anatomic site; knee 31.3%, ankle 28.1%, lower leg 14%, upper leg 11%, foot 6.3%, back 6.3%, upper limb 1.5%, chest wall 1.5%. Lower limb total: 90.1%	PFPS 15.6%, Achilles tendinopathy 7.8%, anterior compartment tendinitis 7.8%, extensor digitorum tendinitis 7.8%, MTSS 7.8%, Quadriceps tendinitis 7.8%, anterior compartment pain 6.3%, ITB 4.7%, quadriceps strain/tear 4.7%, back muscle strain 3.1%, peroneal tendinitis 3.1%, non-specific knee 3.1%, greater trochanteric bursitis 3.1%, extensor hallucis longus tendinitis 3.1%	None, but 72% detrimental effect on performance,	Medical team speak to runner and injury confirmed by history and examination of experienced medical team	19 completed race within cut off time of 8.5 days.
Bishop and Fallon, 1999	Track (grass); 6-day race (time-limited), (Colac, Victoria, Australia)	prospective cohort study	17 (16 men, 1 woman; average age 47)	36 MSKI in 11 runners, injury rate 2.1 injuries/person; all overuse injuries; anatomical site: ankle 36%, knee 19 %	Achilles tendinitis 19%, extensor digitorum longus tendon 14%, PFPS 14%, anterior compartment pain 11%	2 (4.3%); bilateral Achilles tendinitis and quadriceps strain	MSKI defined as affecting performance; interview every 6 h, each injury examined by two experienced physicians	Change of track direction every 2 h; Majority of MSKI presented on day 2 and 3 (75%)
Scheer and Murray, 2011	Off-road (trail); 5-day stage race, 219 km (Al Andalus Ultra Trail, Loja, Spain)	observational, prospective	69 (48 men, average age 46 years; 21 women, average age 40 years)	MSKI: 12 runners (17,4%); 1.8 injuries/competitor. Mostly knee (PFPS). Most medical encounters day 3 and 4.	PFPS 7.2%, Achilles tendinopathy 2.9%, hip (trochanteric bursitis) 1.4%, ultramarathon ankle 1.4%, ankle inversion injury 1.4%, muscle pain (quadriceps 1.4%, tibialis anterior 1.4%)	9 runners did not complete the race; 1 runner (1.4%) because of MSKI (PFPS)	Self-reported by athlete to medical team; medical encounters	Routine medical clinics held twice daily
Krabak et al., 2011	Off-road (trail); 7-day stage race, 240 km (Racing The Planet 4 Desert Series)	prospective	396 (79.2% male, 20.8% female, average age 40)	MSKI 18.2%, mostly minor, during day 3 or 4; 0.71 MSKI/runner. Major MSKI 46.2/1000 runners, 0.8/1000 h; Minor MSKI 670.0/1000 runners, 11.2 per 1000 h. Overall 7.2 injuries/1000 h	MSKI by diagnosis: tendonitis 11.3%, sprain 3.2%, bursitis 1.6%, strain 1.6%, other 4.8% Location: lower extremity 92.6%, mostly The lower leg (35%), ankle (16.8%), knee (13.1%), foot (12.6%),	1.2% race withdrawal	Self-reported medical encounter at checkpoint; MSKI: disability during the race, resulting in medical encounter, minor if able to continue, major if had to withdraw	Data from 4 races (Gobi Desert 2005 and 2006, Sahara Desert, Atacama Desert), 396 runners participated (303 unique individuals)
Graham et al., 2012	Off-road (trail); 7-day stage race, 240 km (Gobi Desert, Mongolia)	prospective	11 (11 men, average age 33 years)	pain, lack of joint mobility (stiffness in knee), Achilles pain, shin pain (similar pattern to previous studies)	N/A	none	Medical assessment twice daily (morning/night) by physician	Abrasion and blisters 100%
McGowan and Hoffman, 2015	Off road (trail); 161 km (Western States Endurance Run, California, United States)	retrospective	63 consultations (of unique individuals), average age 42/43 years across 4 years	Consultations of MSKI 2.6% of all starters	Sprain, strain or tendinitis 0.9%, muscular pain 0.7%, contusion 0.3% (percentages are given in relation to race starters)	0.7% (muscle cramping, sprain, strain or tendinitis)	Self-reported medical encounters at aid stations, retrospective analyses; Pooled data from 4 events (consecutive years);	Total of 8.2% starters sought medical care, most for medical problems (e.g., nausea), including non-competitors (10%)
Vernillo et al., 2016	Off-road (trail); 65 km (Vigolana Trail, Trento, Italy)	prospective	77 (64 men, 13 women, average age 44 years)	MSKI 32.8%, all minor; injury rate: 614 per 1000 runners; 4285 per 1000 h,	Plantar fasciitis 28.6%, ankle sprain 28.6%, knee sprain 14.3%, thigh sprain 14.3%, Achilles tendinopathy 7.1%, neck/cervical sprain 7.1%	none	Self-reported medical encounter post-race via questionnaire; MSKI: disability during the race, resulting in medical encounter, minor if able to continue, major if had to withdrawal	No major injuries
Dawadi et al., 2020	Off-road (trail); 7-stage 212 km (Manaslu trail race, Himalaya)	retrospective	100 (60 men, 40, women)	MSKI 17%; 170 injuries/1000 athletes or 1.2 cases per 1000 km run	Most commonly ankle sprain; one case of subluxated distal phalanx of finger	none	Self-reported to medical team	Poole data from 3 consecutive events; no data on specific MSKI/incidence; competition at altitude

**TABLE 2:** Studies investigating musculoskeletal injuries (MSKI) during training.

Study	Study design	Participants	General observations	Specific MSKI	Additional observation
Micklesfield et al., 2007	retrospective, questionnaire survey, at registration prior to Two Ocean Ultra (56 km), South Africa, by trained interviewer, random prior athlete selection	276 ultra runners, (all female), average age 39 years	Examined only bone stress injuries in female ultra-runners	Bone stress injury 21%	No anatomical site of bone stress given. No distinction between bone stress injury and stress fracture.
Hoffman and Krishnan, 2014	retrospective, questionnaire, self-reported	1212 ultra runners (824 men, 399 women), average age 42.3 years	Injuries during last 12 months (training/competition) ULTRA study	Knee issues 24%, back injuries 12.4%, ITB 15.8, hamstring strain 11.8%, calf strain 13.1%, Achilles tendinitis or tear 10.8%, ankle sprain 10.8%, plantar fasciitis 10.6%, other foot and ankle injuries 4.5%, bunion 2.5%, stress fracture foot 3.4%, metatarsalgia 3.1% lower leg or ankle tendinitis 9.2%, pelvis or hip issues 3.7, hip flexor strain 8.7, fractures not involving the extremities 1%, upper extremity 1.4%, femur/hop stress fracture 0.5%, tibia/fibula stress fracture 1.9%, other lower leg injuries 1.5%,	
Malliaropoulos et al., 2015	retrospective, epidemiological questionnaire, convenience sample of ultra-trail runners	40 ultra runners (36 men, 4 women), average age 39.4 years	Total of 134 injuries, 90% at least one injury, mean numbers 3.38 injury/individual; 82.2% overuse; 17.7% during competition.	Diagnosed injuries (43): 22% overuse bone stress, spinal disk/low back 14%, ITB 16%, meniscal injury 14%, hamstring 12%, Achilles tendinopathy 7%, plantar fasciitis 7%, Morton’s neuroma 5%, tibiofibular joint injury 2%, Adductor tendinopathy 2%; Injured areas anatomical site: 42.5% lower back, 40% knee	
Scheer et al., 2020d	retrospective survey study via questionnaire	78 runners (65 men, 13 women), average age 38.0 years	Examined adults, that participated in UER as a youth athlete (<19 years of age). Participated at first ultra at average age of 16.1 years. MSKI 23.1 %; stress fractures 6.4; 1.9 injuries/athletes	21.4 % knee pain, 17.9% ankle sprain, 14.2% ITB, 10.7% tibial stress fracture, 3.6% each: ankle tendinopathy, Achilles tendinopathy, hip flexor strain, foot stress fracture, hamstring strain, plantar fasciitis, MTSS, Morton’s neuroma, bursitis.	Lifetime prevalence of stress fractures of adults that started running ultras as youth athletes: 14.1%

### Anatomical Regions and Specific Diagnoses

#### Hip

The incidence of hip injuries during multi- day UER events was around 3.8% (Krabak et al., 2011), with the iliotibial band the most commonly affected structure. Iliotibial band syndrome (ITBS) is generally an overuse injury of the connective tissue around the lateral thigh and/or knee (Taunton et al., 2002; Fredericson and Wolf, 2005; Fredericson and Weir, 2006; Strauss et al., 2011) with a prevalence of between 14.2% (Malliaropoulos et al., 2015) and 15.8% in adult UER (Hoffman and Krishnan, 2014), and 14.2% in youth UER (Scheer et al., 2020d). The incidence on race day during a 1005 km continuous road race was lower with an incidence of approximately 4.7% (Fallon, 1996). Hip injuries encountered during race day were typically secondary to a bursitis [psoas bursitis 11.1% (Hutson, 1984), greater trochanteric bursitis (1.4%–.1% (Fallon, 1996; Scheer and Murray, 2011)], while injuries during training often included hip flexor strains (3.6–8.7%) (Scheer and Murray, 2011; Hoffman and Krishnan, 2014) and adductor tendinopathy (2%) (Malliaropoulos et al., 2015).

#### Upper Leg

Injuries to the structures of the upper leg were mostly encountered during competition, and include injuries such as quadriceps muscle pain (1.4%) (Scheer and Murray, 2011), quadriceps muscle strain or tear [ranging from 4.7% (Fallon, 1996) to 14.3% (Vernillo et al., 2016)], and quadriceps tendinitis [7.8% (Fallon, 1996)]. During training hamstring muscle strains have been described in 3.8% of youth athletes (Scheer et al., 2020d) and 11.8% in adult UER (Hoffman and Krishnan, 2014).

#### Knee

The knee is one of the regions most frequently injured, with incidences during competition ranging between 13.1% (Krabak et al., 2011) to 31.3% (Fallon, 1996). Diagnosis included patellar tendinitis/tendinopathy (18.5%) (Hutson, 1984), knee sprains (14.3%) (Vernillo et al., 2016) or other non-specific knee pains (3.1%) (Fallon, 1996). Patella femoral joint disorders or patella femoral pain syndrome (PFPS) were frequent diagnoses and the incidence ranged between 7.2–15.6% (Fallon, 1996; Scheer and Murray, 2011). During training the prevalence of knee injuries was between 21.4% in youth athletes (Scheer et al., 2020d) to 24% in adult UER (Hoffman and Krishnan, 2014). Meniscal injuries were also prevalent (12%) (Malliaropoulos et al., 2015).

#### Lower Leg

The lower leg was frequently injured during competition ranging from 14% (Fallon, 1996) to 35.0% for multi-day, multi-stage UER events (Krabak et al., 2011) and included a variety of pathologies, such as medial tibial stress syndrome (MTSS) and chronic exertional compartment syndrome (CECS).

Medial tibial stress syndrome [sometimes called shin soreness, shin splints, tibial stress syndrome, medial tibial periostitis and medial tibial traction periostitis (Moen et al., 2009; Reshef and Guelich, 2012)] had an incidence of between 7.8% (Fallon, 1996) and 10.1% (Hutson, 1984) in competition and a prevalence of 3.6% in youth runners (Scheer et al., 2020d).

Chronic exertional compartment syndrome affected most commonly the anterior compartment, that contain the dorsiflexor muscles of the tibialis anterior, extensor digitorum longus, and extensor hallucis longus muscles (Fraipont and Adamson, 2003; George and Hutchinson, 2012). Diagnosis included tibialis anterior muscle pain (1.4%) (Scheer and Murray, 2011), anterior compartment pain ranging from 6.3% (Fallon, 1996) to 11% (Bishop and Fallon, 1999), and anterior compartment tendinitis (7.8%) (Fallon, 1996). Plantar flexor tendinitis/peroneal tendinitis of the lateral compartment was described in 3.1% of UER (Fallon, 1996). Gastrocnemius muscle strain occurred in 3.7% during competition (Hutson, 1984) and 13.1% (Hoffman and Krishnan, 2014) during training.

#### Ankle

The ankle is another frequently injured anatomical site and some investigations cite this as the most common site of injury in UER during competition, however, there was a wide range of incidences of between 16.8% (Krabak et al., 2011) to 36% (Bishop and Fallon, 1999). Tendinitis of the dorsiflexors of the foot was the most frequent diagnosis (29.6%) (Hutson, 1984) and called ‘ultramarathon ankle,’ a relatively specific injury to UER (Hutson, 1984; Fallon, 1996; Bishop and Fallon, 1999; Scheer and Murray, 2011). Repetitive plantar and dorsiflexion as observed during prolonged running, may cause a peritendinitis/tenosynovitis of the tendons passing under or adjacent to the extensor retinaculum of the ankle (Hutson, 1984; Fallon, 1996). Other causative factors include excessive pressure on the dorsum of the ankle due to tight fitting shoes, over-pronation, running on hard surfaces, and overstriding (Hutson, 1984; Fallon, 1996). The incidence varied and was as low as 1.4% (Scheer and Murray, 2011) and reached 29.6% (Hutson, 1984). More specifically, depending which dorsiflexor of the foot was affected the incidence varied [e.g., extensor digitorum longus 7.8% (Fallon, 1996) to 14% (Bishop and Fallon, 1999); or extensor hallucis longus 3.1% (Fallon, 1996)].

Achilles tendinopathy was another frequent MSKI with a wide range of incidences during competition, from 2.9% during a 5 day multi stage UER event (Scheer and Murray, 2011) to 7.1% in a 65 km trail race (Vernillo et al., 2016) and 7.8% in a 1005 km road UER (Fallon, 1996). The highest incidence was reported in UER events on a track with incidences of between 18.5% and 19% (Hutson, 1984; Bishop and Fallon, 1999). In training studies Achilles tendinopathy was observed in between 7% (Malliaropoulos et al., 2015) and 10.8% (Hoffman and Krishnan, 2014), and 17.9% in youth athletes (Scheer et al., 2020d). Ankle sprains were frequently observed in UER trail races (28.6%) (Vernillo et al., 2016), but were less frequent in multi-day UER events (1.4%) (Scheer and Murray, 2011). During training prevalence of ankle sprains ranged from 10.8% in adult UER (Hoffman and Krishnan, 2014) to 17.9% in youth UER (Scheer et al., 2020d). Tibio-fibular joint injuries were observed in 2% of UER (Malliaropoulos et al., 2015).

#### Foot

Foot injuries were also very common injuries in UER, with a wide range of incidences between 6.3% (Fallon, 1996) and 12.6% (Krabak et al., 2011). Plantar fasciitis was reported in 28.6% in trail runners during a competition (Vernillo et al., 2016), while numbers in training studies are lower, with a prevalence ranging from 7% (Malliaropoulos et al., 2015) to 10.6% (Hoffman and Krishnan, 2014) in adult UER and 3.6% in youth runners (Scheer et al., 2020d). Other pathologies included metatarsalgia (3.1%), bunion (2.5%) (Hoffman and Krishnan, 2014) and Morton’s neuroma (3.6%) (Scheer et al., 2020d).

### Bone Stress Injuries of the Lower Limbs

Bone stress injuries can vary in severity, with early injuries demonstrating periosteal edema and/or bone marrow edema on radiological examination, with more severe injury showing a stress fractures with radiological evidence of a fracture line (Tenforde et al., 2016).

Stress fractures are usually fatigue fractures that develop through overuse on healthy bone (Harrast and Colonno, 2010; Pegrum et al., 2012; Krabak et al., 2019; Tenforde et al., 2019). None of the studies during competition described stress fractures, however, this may be difficult to diagnose clinically. Prevalence is high in training, ranging from 6.4% in youth UER (Scheer et al., 2020d) to 22% in adults (Malliaropoulos et al., 2015). Bone stress was particularly high among female athletes (21%) (Micklesfield et al., 2007), however, it is not clear from the study if these were early bone stress injuries or actual stress fractures. Bone stress injuries in female athletes were associated with increased energy expenditure, and associated with inadequate nutrition as seen in relative energy deficiency in sport (RED-S) (Micklesfield et al., 2007; Tiller et al., 2021). Prevalence of stress fractures in adult UER were observed in different anatomical regions, such as the foot (3.4%), tibia (1.9%), and femur/hip (0.5%) (Hoffman and Krishnan, 2014) and in youth athletes tibia (10.7%), and foot (3.6%) (Scheer et al., 2020d).

#### Low Back

Back injuries were less frequently encountered in UER competitions (1.4%–6.3%) (Fallon, 1996; Krabak et al., 2011) and included back muscle strains (3.1% (Fallon, 1996). Back pain was more commonly encountered during training, with a prevalence of between 12–14% (Hoffman and Krishnan, 2014; Malliaropoulos et al., 2015) but not all may have been related to running, but other activities of daily life (Malliaropoulos et al., 2015).

#### Upper Body

Upper body injuries were also not commonly described in UER competitions with an incidence of up to 3.6% (Fallon, 1996; Krabak et al., 2011) and included the chest wall (1.5%) (Fallon, 1996) or neck sprains (7.1%) (Vernillo et al., 2016).

### MSKI by Type of UER Event

#### MSKI During Time-Limited UER on Track

Though research is limited, two studies compared MSKI during a 6 day time-limited UER events on a track (Hutson, 1984; Bishop and Fallon, 1999). One race was held on a tartan track with a change of running direction every 12 h (Hutson, 1984), and competitors averaged 936 km over the 6 day period. The main anatomical site of injury was the ankle (48%), followed by the knee (26%), involving the extensor muscle-tendon structure of the ankle (41%) and the knee (26%), tendonitis of foot dorsiflexors (30%) and Achilles tendonitis (19%). In comparison, the other time-limited UER event was held on a grass track, with the mean distance covered of 836 km (Bishop and Fallon, 1999) and the ankle was the anatomical site most frequently injured (36%), followed by the knee (19%). The extensor -muscle-tendon complex of the ankle (25%) and knee (22%) was less commonly affected, as well as tendonitis of the foot dorsiflexors (14%). The occurrence of Achilles tendonitis (19%) was similar (Bishop and Fallon, 1999).

#### MSKI During Multi-Stage, Multi-Day UER Events in Off-Road Terrain

Four studies examined MSKI during multi-day UER events on off-road terrain (Krabak et al., 2011; Scheer and Murray, 2011; Graham et al., 2012; Dawadi et al., 2020). They were all similar and comparable in length, and duration, of between 5 to 7 days, covering 219–240 km but differed in environmental conditions, with 3 of them in hot or desert environment (Krabak et al., 2011; Scheer and Murray, 2011; Graham et al., 2012) and one in cold environment, at altitude (Dawadi et al., 2020). Although two studies (Graham et al., 2012; Dawadi et al., 2020) did not provide specific diagnosis or incidences of MSKI, all injuries were minor and patterns observed were comparable to previous studies (Graham et al., 2012). The rate of MSKI were similar in three of the studies (17.0, 17.4, and 18.2%, respectively), varying between 0.7 injuries/runner and 1.8 injuries/runner (Krabak et al., 2011; Scheer and Murray, 2011; Dawadi et al., 2020). MSKI injury rates were reported in two studies but used different denominators, with a desert race reporting (minor MSKI 670.0 injuries/1000 runners; major MSKI 11.2 injuries/1000 h; overall 7.2 injuries/1000 h) (Krabak et al., 2011) and a race in the Himalaya (170 injuries/1000 runners or 1.2 cases per 1000 km) (Dawadi et al., 2020). Most MSKI were minor, occurred during the middle part of the race (around day 3 and 4) and affected the lower limbs, mainly lower leg, knee, ankle and foot (Krabak et al., 2011; Scheer and Murray, 2011). Two study showed a predominance of the lower leg/foot/ankle, whereas the other reported injuries mostly to the knee and ankle (Krabak et al., 2011; Scheer and Murray, 2011; Dawadi et al., 2020). The number of runners withdrawing from the race because of MSKI was also very similar (1.2% vs 1.4%) (Krabak et al., 2011; Scheer and Murray, 2011) with no withdrawals because of MSKI in the other two studies (Graham et al., 2012; Dawadi et al., 2020).

#### MSKI During Continuous UER Events

Three studies examined single, continuous UER events of different terrain and distance. While two races were held on off-road terrain, with distances of 65 km and 161 km (McGowan and Hoffman, 2015; Vernillo et al., 2016), the third was a road race over 10005 km (Fallon, 1996). The study examining the 161 km race was a retrospective analysis of medical encounters of four past edition of the same race, but did not provide specific anatomical distribution and diagnosis of MSKI, but 2.6% of all starters sought medical advice for MSKI, mostly for sprains, strains, tendinitis or muscular pain (McGowan and Hoffman, 2015). Injury rates for the 65 km UER event where high, with 614 injuries/1000 runners and 4285 injuries/1000 h (Vernillo et al., 2016). While MSKI were present in 32.8%, all were minor in nature, predominantly affecting the foot (plantar fasciitis), ankle and knee, with none of the runners having to withdrawal because of MSKI (Vernillo et al., 2016). Similarly, in the 10005 km road event, no runner had to withdrawal due to major MSKI, however, almost three quarters noticed a negative effect on performance (Fallon, 1996). Over 90% of MSKI affected the lower leg, with the main anatomical site the knee, ankle and foot (Fallon, 1996).

#### MSKI During Training

Four studies examined MSKI retrospectively during training, with longer observational periods via questionnaires (Micklesfield et al., 2007; Hoffman and Krishnan, 2014; Malliaropoulos et al., 2015; Scheer et al., 2020d), whereas one looked specifically at bone stress in female UER athletes (Micklesfield et al., 2007) and one at youth athletes (Scheer et al., 2020d). Bone stress in females was common and comparable to another study with mostly male athletes (21% vs 22%) (Micklesfield et al., 2007; Malliaropoulos et al., 2015), but less than in runners that started UER during their youth (14.1%) (Scheer et al., 2020d). Injury rate was between 1.9 and 3.4 injuries/athlete (Malliaropoulos et al., 2015; Scheer et al., 2020d), with the majority being overuse injuries, mainly affecting the knee, ankle and ITB (Hoffman and Krishnan, 2014; Malliaropoulos et al., 2015; Scheer et al., 2020d).

## Discussion

The aims of this scoping review were therefore: (1) to examine the current evidence of MSKI, providing a synthesis of the most common MSKI by anatomical region and specific diagnosis; (2) categorize MSKI by type of UER activity (competition: time-limited; multi-stage; continuous UER events and training); (3) describe knowledge gaps in the literature and provide advice on potential further research. The main findings of our review were that MSKI were mostly overuse injuries, predominantly affecting the lower limbs with different injury patterns and diagnosis between different types of UER activities.

### MSKI by Anatomical Region and Diagnosis

The literature review identified 13 studies that reported on MSKI in UER. There is agreement across all studies that MSKI in UER are common, mostly overuse in nature and predominantly affecting the lower limbs (Hutson, 1984; Fallon, 1996; Bishop and Fallon, 1999; Krabak et al., 2011; Scheer and Murray, 2011; Graham et al., 2012; Hoffman and Krishnan, 2014; Malliaropoulos et al., 2015; McGowan and Hoffman, 2015; Vernillo et al., 2016; Dawadi et al., 2020; Scheer et al., 2020d). The main anatomical sites for MSKI are the lower leg, knee, ankle and foot, with the main diagnosis of MTSS, Achilles and patella tendinopathy, PFPS, ankle sprains, plantar fasciitis, ultramarathon ankle and bone stress injuries (Hutson, 1984; Fallon, 1996; Bishop and Fallon, 1999; Micklesfield et al., 2007; Krabak et al., 2011; Scheer and Murray, 2011; Graham et al., 2012; Hoffman and Krishnan, 2014; Malliaropoulos et al., 2015; McGowan and Hoffman, 2015; Vernillo et al., 2016; Dawadi et al., 2020; Scheer et al., 2020d). Most injuries are generally minor in all types of UER events (Fallon, 1996; Bishop and Fallon, 1999; Krabak et al., 2014), but nevertheless can affect performance or lead to race withdrawal (Bishop and Fallon, 1999; Scheer and Murray, 2011; Graham et al., 2012; Krabak et al., 2014; Vernillo et al., 2016).

### Definition of MSKI

One of the challenges in comparing MSKI across the different studies was, that no uniform definition of MSKI was used. Some studies provided no specific definitions of MSKI (Scheer and Murray, 2011; Graham et al., 2012; McGowan and Hoffman, 2015; Dawadi et al., 2020), whilst others defined MSKI as a disability resulting in a medical encounter (Krabak et al., 2011; Vernillo et al., 2016), affecting performance (Bishop and Fallon, 1999), based on a diagnosis of a health care provider, or via self-assessment (Micklesfield et al., 2007; Hoffman and Krishnan, 2014; Malliaropoulos et al., 2015; Scheer et al., 2020d). Classifying and using a standard reporting systems of MSKI in UER is important, as lack of a standardized definition affects the rates of injuries and hinders comparison between studies (Kluitenberg et al., 2015; Yamato et al., 2015a, b). Further, most studies include small sample sizes, and therefore possibly influencing the rate of diagnosis, as with small changes in a particular diagnosis this can lead to large percentage differences. This may also be of particular interest in diagnoses that are made clinically during competitions, such as stress fractures or CECS. CECS is an overuse injury that presents with increased pressure within one of the compartments of the lower leg, that can lead to ischemia, decreased tissue perfusion and pain (Fraipont and Adamson, 2003; George and Hutchinson, 2012). The gold standard for diagnosis CECS is with compartmental pressure testing (Fraipont and Adamson, 2003; George and Hutchinson, 2012). This is not feasible during competition and diagnosis presented in the studies are made on clinical examination and suspicion. Therefore, some MSKI may be either underrepresented (e.g., stress fractures) or overrepresented (e.g., CECS) in small samples and not reflect the true incidence. Interestingly, none of the training studies reported on CECS, but a high number of stress fractures were observed, ranging from 6–22% (Micklesfield et al., 2007; Malliaropoulos et al., 2015; Scheer et al., 2020d).

Future studies therefore should consider examining MSKI on larger populations, either in larger races, pooling data from similar events or over several editions of the same event, to provide a better understanding of the incidence of MSKI. Similarly, it is important to examine different type of events as the limited research has shown, that MSKI are distributed differently depending on type of UER.

### MSKI Comparing Different UER Events

During two 6 day time-limited UER events on the track, with an average distances of 836–936 km, the main anatomical site of injury was the ankle (36–48%) and the knee (19–26%), with tendonitis of foot dorsiflexors (25–41%) and Achilles tendonitis (19%) as the most frequent diagnosis (Hutson, 1984; Bishop and Fallon, 1999). Running on a loop-course of track may put additional stress on one particular anatomical side, however, in both studies injuries were evenly distributed between the left and right limb (Hutson, 1984; Bishop and Fallon, 1999), demonstrating that a changing of the running direction while running on a track, may help in preventing lower limb injuries (Gajda et al., 2020). Although, the lower limb was also commonly injured during multi-day UER events (ranging between 219 km–240 km) the distribution of MSKI differed [lower leg (35%), ankle (16.8%), knee (13.1%), and foot (12.6%)], with 0.2–1.8 injuries/runner and overall incidence of 7.2 injuries/1000 h or 1.2 injuries/1000 km (Krabak et al., 2011; Scheer and Murray, 2011; Graham et al., 2012; Dawadi et al., 2020). However, by comparison the overall distance covered in the same time period was very different (219–240 km vs. 836–936 km), that may be result in different injury patterns.

Three studies examined continuous UER events, ranging from 65 km to 10005 km (Fallon, 1996; McGowan and Hoffman, 2015; Vernillo et al., 2016). The shortest (65 km) race reported the highest number of MSKI (32%), with injury rates of 614 injuries/1000 runners and 4285 injuries/1000 h, with the main anatomical site, the foot (28.6%), ankle (28.6%) and knee (14.3%) (Vernillo et al., 2016). This represents the highest incidence of any UER event, possibly related to the off-road (trail) environment where the race was held, with significant elevation change and prolonged uphill and downhill running sections. Ankle and knee injuries were mostly diagnosed as sprains, possibly more related to acute injuries than true overuse injuries, although no further clinical assessment or diagnostic criteria are provided (Vernillo et al., 2016). But it is also possible that due to the short nature of the race, the running speed was comparatively faster compared to the other races, making injuries more likely. During a 1005 km road race, overuse MSKI were common with over 90% affecting the lower leg (31.3% knee, 28.1% ankle) (Fallon, 1996). The 161 km does not provide specific anatomical distribution and diagnosis of MSKI, but overall 2.6% of all starters sought medical advice for MSKI, mostly for sprains, strains, tendinitis or muscular pain (McGowan and Hoffman, 2015).

### MSKI During Training

Although training studies also showed a predominance for lower limb MSKI, bone stress and stress fractures were commonly reported in contrast to data from competition, ranging from 6–21% (Micklesfield et al., 2007; Malliaropoulos et al., 2015; Scheer et al., 2020d), likely due to the longer observational periods and better diagnostic possibilities. Similarly, back injuries are rarely reported during competition but during training this varies between 12.4%–14%, although it may not always be directly related to running but to other activities of daily living (Hoffman and Krishnan, 2014; Malliaropoulos et al., 2015). Injury rates during training were between 1.9 and 3.4 injuries/athlete (Malliaropoulos et al., 2015; Scheer et al., 2020d), which is higher compared to injury rates during most competitions (0.7–2.1 injuries/runner) (Bishop and Fallon, 1999; Krabak et al., 2011; Scheer and Murray, 2011), possibly demonstrating the effects of continued large training loads and/or inadequate rest days. The highest injury rates were observed in a 65 km off-road UER event with 4.3 injuries/runner (Vernillo et al., 2016), suggesting that the demand of short and faster off-road events may have higher incidences of MSKI.

### Practical Considerations

These findings may be of interest to a variety of practitioners. Firstly, medical practitioners and health care providers may be able to plan medical provisions when attending UER competitions. There is guidance on how to provide medical care at UER events (Hoffman et al., 2014), however, given the increased risk of MSKI during shorter, off-road UER events, these provisions may need to be adapted and/or increased. Similarly, medical practitioners may be able to treat training injuries more appropriately, especially considering the increased risk of bone stress injuries and back injuries during training. Secondly, coaches and athletes may benefit of the knowledge of the distribution of injuries and type of UER activity and plan their training program accordingly to try and avoid these overuse injuries.

### Future Research Directions

Sports scientist and researchers may benefit from the synthesis of the available evidence and suggestions for future reference, helping them design further studies to reduce the knowledge gap. Examining different UER events, environments and surfaces may therefore also help to gain a better understanding of the underlying mechanisms of MSKI. Prior running experience and training history of the athlete as well as prior personal injury history may also be important and should be investigated. This may help to evaluate the impact of MSKI on race performance and withdrawal, as MSKI can lead to performance decrement (Fallon, 1996) and race withdrawal in 0.7%–4.8% of runners (Hutson, 1984; McGowan and Hoffman, 2015). Further, most studies include both sexes and sex difference have not been examined separately, however, it may be of interest as in non-UER females have more bone stress injuries while male runners more Achilles tendon injuries, although overall injury rates in non-UER athletes are similar (20.8 injuries/1000 females and 20.4 injuries/1000 males) (Hollander et al., 2021). Generally, there is less research in female UER, and it would be important to examine sex differences, as key aspects of female athlete physiology warrant careful consideration, e.g., RED-S that may lead to physiological impairments resulting in an increased risk of bone stress injuries (Tiller et al., 2021). Additionally, age differences in MSKI have not been examined, which is another important aspect, especially if UER could lead to negative long-term health effects on the musculoskeletal system.

Examination of risk factors leading to MSKI in UER is another important aspect as there seems to be a U-shaped pattern between the running distance and the time-loss injury (Kluitenberg et al., 2015). Especially in UER, that requires large training volumes this will be an important aspect, as well as strategies to reduce MSKI. MSKI may be reduced through online tailored advice and this has been observed in trail runners participating in distances of between 15–62 km (Hespanhol et al., 2018), however, if this is particularly applicable to UER is not known but an interesting aspect to investigate further. It may also be interesting to explore how long athletes have to refrain from UER after a MSKI or if they part take in other sports (e.g., cycling) either during rehab or stop UER altogether.

## Conclusion

Musculoskeletal injuries in UER are common, mostly affecting the lower limb and are of overuse in nature. MSKI differ between competition and training, with multistage events, predominantly affecting the lower leg (medial tibial stress syndrome), foot, knee (patella femoral pain syndrome (PFPS), while timed events mainly affect the ankle (tendinitis of the foot dorsiflexors, Achilles tendinopathy), and knee (patellar tendinopathy). Short continuous UER events off-road have the highest incidence of MSKI, mainly affecting the foot (plantar fasciitis), ankle (ankle sprain), whereas in long continuous UER the main anatomical site of injury is the knee (PFPS), and ankle. During training the back, knee and bone stress injuries are common. Future considerations include examining MSKI in different UER events, environments and surfaces, and on larger study populations. Establishing risk factors, examining sex differences and using a standard reporting system of MSKI in UER are also important.

## Author Contributions

VS performed conception and design of the study, literature search and analysis, and manuscript writing and editing. BK performed literature search and analysis and manuscript writing and editing. Both authors contributed to the article and approved the submitted version.

## Conflict of Interest

The authors declare that the research was conducted in the absence of any commercial or financial relationships that could be construed as a potential conflict of interest.
